# Impact of Early Tracheostomy on Weaning From Ventilation and Sedation in COVID-19 Pregnant and Early Postpartum Patient: Two Case Reports

**DOI:** 10.7759/cureus.29633

**Published:** 2022-09-26

**Authors:** Edin Karic, Hassan Mitwally, Lolwa M Alansari, Adel Ganaw, Mohamed O Saad, Abdulhamid Azhaghdani

**Affiliations:** 1 Critical Care Medicine, Hamad Medical Corporation, Al Wakra, QAT; 2 Pharmacy, Hamad Medical Corporation, Al Wakra, QAT; 3 Obstetrics and Gynecology, Hamad Medical Corporation, Al Wakra, QAT

**Keywords:** sedation, weaning from mechanical ventilator, covid-19, pregnancy, early tracheostomy

## Abstract

Pregnant women are at high risk of coronavirus disease 2019 (COVID-19) complications, including acute respiratory distress syndrome (ARDS) and the need for mechanical ventilation. There is no literature on the optimal strategy for the management of difficult-to-wean pregnant and early postpartum patients.

We report two cases of pregnant women with COVID-19 pneumonia and ARDS, who required mechanical ventilation and high doses of analgesia, and sedation with neuromuscular blocking agents to facilitate ventilation and oxygenation. Both patients had a tracheostomy procedure to facilitate weaning from mechanical ventilation and sedation. Shortly after tracheostomy, sedation and analgesia, along with ventilatory support were weaned off. Both patients were discharged home. These cases propose early tracheostomy as a strategy to facilitate weaning from mechanical ventilation and sedation in pregnant and early postpartum patients.

## Introduction

Since the World Health Organization (WHO) has defined the coronavirus disease 2019 (COVID-19) outbreak as a pandemic, COVID-2019 has triggered several waves in most countries around the globe. Pre-existing comorbidities, non-white ethnicity, chronic hypertension, pre-existing diabetes, high maternal age, and high body mass index are risk factors for severe COVID-19 outcomes in pregnancy, and pregnant women with COVID-19 are at higher risk for admission to the intensive care unit (ICU) and mechanical ventilation (MV) than non-pregnant women of reproductive age [1]. Additionally, during the second wave of the COVID-19 pandemic, pregnant and peripartum women experienced more severe illness than in the first wave [2]. A number of case reports of severe COVID-19 pregnant women requiring intensive care treatment and MV have been reported [3-6].

The recommendations for analgesia/sedation in mechanically ventilated pregnant patients are generally similar to nonpregnant individuals [7]. Many intubated patients with severe COVID-19 infection require high doses of analgesia/sedation with or without a neuromuscular blocking agent (NMBA) [8]. This usually increases the risk of delirium and impacts weaning from ventilation, length of stay, mortality, and costs [9-11].

Weaning from MV is the process of withdrawing ventilator support, which usually starts with spontaneous breathing trials (SBT). Weaning failure is defined as either the failure of SBT or the need for reintubation within 48 hours following extubation [12,13]. Weaning from sedation is the process of liberation from any sedative agent, which usually starts before or goes in line with weaning from ventilation. The application of weaning protocols for sedation and ventilation in ICU is a daily routine [14].

The conversion from an endotracheal tube to tracheostomy significantly improves the measured values of weaning parameters in difficult-to-wean patients [15]. Tracheostomy appears to facilitate weaning from sedation. The short duration of MV after tracheostomy suggests that sedation requirements and ventilator dys-synchrony may be primary barriers to weaning these patients from the ventilator [16]. A systematic review, which included eight randomized control trials with around 2000 patients, analyzed the timing of tracheostomy, suggesting early tracheostomy might be superior to late tracheostomy [17].

During the second wave, we observed that most pregnant and postpartum patients required excessive doses of multiple analgesic and sedation agents with or without NMBA to facilitate ventilation and oxygenation as life-saving management in severe acute respiratory distress syndrome (ARDS). To our knowledge, there is no literature that covers the regimen of sedation and the ideal time of tracheostomy in pregnant, critically ill COVID-19 patients. In these two case reports, we shared our experiences of how (early) tracheostomy in this patient population may impact the weaning from ventilation and sedation.

## Case presentation

Case one

A 32-year-old, gravida three (week 29 + three), para two with Middle Eastern background, with no chronic illness, hospitalized on the day of COVID-19 positive PCR test due to pneumonia. She was on metformin at home for gestational diabetes. The patient was admitted to the ICU, with a SOFA score of 3, after one week due to severe respiratory distress and deterioration on the obstetric ward with 91% oxygen saturation (SpO^2^) on 15 liters of oxygen on a non-nonrebreathing mask (NRBM) with abnormal arterial blood gases (Table 1). Vital signs upon ICU admission: blood pressure (BP) 118/58 mmHg, heart rate (HR) 112/min, respiratory rate (RR) 31/min, and temperature 37.2 °C.

**Table 1:** Arterial blood gases analysis upon ICU admission pO_2_ – Partial pressure of oxygen, pCO_2_ – Partial pressure of carbon dioxide, BE – Base excess, pH – Potential of hydrogen

Parameter	Value	Normal range
pH	7.33 ↓	7.35 – 7.45
pO_2_	72 mmHg	65 – 105 mmHg
pCO_2_	22 mmHg ↓	35 – 45 mmHg
BE	-14.6 mmol/L ↓	-2 to +2 mmol/L
Bicarbonate	11.4 mmol/L ↓	22 – 26 mmol/L

**Figure 1:**
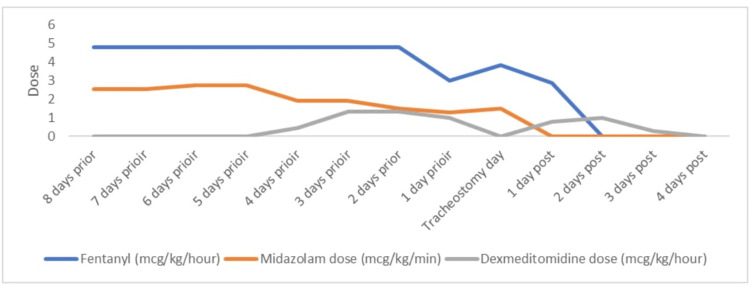
Tracheostomy effect on sedation and analgesia weaning

After admission, a high flow nasal cannula (HFNC) with 30 liters flow rate and 65% fraction of inspired oxygen (FiO_2_) was applied, and the patient got respiratory stabilized. In the evening of the next day, she suddenly deteriorated with desaturation and severe respiratory distress. Following the discussion in the multidisciplinary team, an emergency cesarean section (CS) was performed. Thereafter, the patient remained respiratory and hemodynamically stable and was transferred back to ICU on MV. The obstetric team regularly followed up with the patient. The ensuing two weeks spent at the ICU were accompanied by cytokine storm, hospital-acquired pneumonia (HAP), ARDS, and consequently difficult and prolonged weaning. COVID-19 infection, HAP, and ARDS were treated as per local protocol considering the international guidelines (Table 2). Weaning from sedation and accordingly from ventilation was the main challenge. The patient required simultaneously up to four analgesic and sedative agents at a high dose (Figure 1) plus NMBA cisatracurium up to 3 mcg/kg/min to maintain oxygenation and ventilation. On day 20 of ICU admission, when tracheostomy was applied, she required continuous infusions of fentanyl 5 mcg/kg/hour, midazolam 3 mcg/kg/min, and cisatracurium 2 mcg/kg/min. Ventilator settings on the day of tracheostomy were: adaptive pressure ventilation-controlled mechanical ventilation (APV-CMV), RR 33/min, tidal volume 350 mL, positive end-expiratory pressure (PEEP) 10 cmH_2_O, FiO_2_ 45%. The bedside percutaneous dilatative tracheostomy under bronchoscopy guidance was performed without any complications, and tracheostomy tube size 8 was inserted. COVID-19 PCR status a day before the procedure was inconclusive. During the next 48 hours, most analgetic and sedative agents have been stopped (Figure 1), except dexmedetomidine, which reached up to 1.5 mcg/kg/hr and was stopped 72 hours after tracheostomy. The weaning from the ventilator went smoothly, and the patient was decannulated 10 days after the tracheostomy. On the following day, she was transferred to the ward after 30 days of ICU stay and discharged home after two more days, with 38 days of hospital stay. The baby has been discharged home with the mother in good condition. Follow-up by primary care physicians in the next two months did not show any issue.

**Table 2:** COVID-19, HAP and ARDS treatment HAP - hospital-acquired pneumonia, ARDS - acute respiratory distress syndrome

Drug	Dose	Duration
Ceftriaxone	2 grams, daily	7 days
Azithromycin	500mg, daily	3 days
Enoxaparine	40 mg, twice daily	25 days
Dexamethason	8 mg, daily	10 days
Hydrochloroquine	400 mg, daily	4 days
Lopinavir/Ritonavir	200/50 mg, twice daily	4 days
Remdesivir	1^st^ day 200 mg, then 100 mg, daily	5 days
Tocilizumab	400 mg, once	on two occasions
Meropenem	2 g, q8hr	7 days

Case two

A 33-year-old, gravida three (week 23 + two), para two, with an Asian background, with no chronic illness or risk factors, was hospitalized on the day of COVID positive PCR test due to pneumonia. The patient was admitted to the ICU, with the SOFA score of 4, after one week, due to severe respiratory distress and deterioration in the emergency department, with 91% SpO_2_ on 15 liters of oxygen on NRBM with abnormal arterial blood gases (Table 3). Vital signs upon ICU admission: BP 102/64 mmHg, HR 99/min, RR 30/min, and temp. 37.2 °C.

**Table 3:** Arterial blood gases analysis upon ICU admission pO_2_ – Partial pressure of oxygen, pCO_2_ – Partial pressure of carbon dioxide, BE – Base excess, pH – Potential of hydrogen

Parameter	Value	Normal range
pH	7.42	7.35 – 7.45
pO_2_	67 mmHg	65 – 105 mmHg
pCO_2_	26 mmHg ↓	35 – 45 mmHg
BE	-7.4 mmol/L ↓	-2 to +2 mmol/L
Bicarbonate	17.1 mmol/L ↓	22 – 26 mmol/L

**Figure 2:**
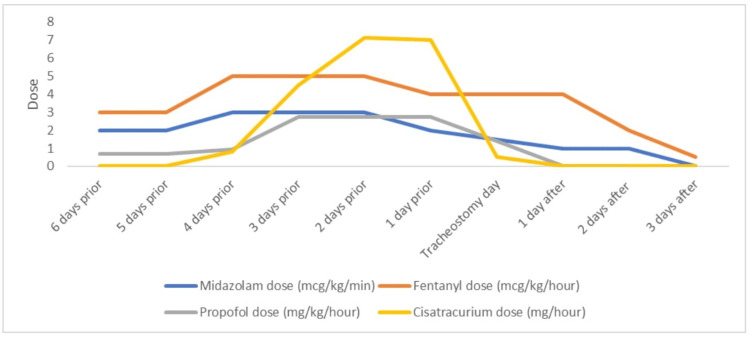
Tracheostomy effect on sedation, analgesia, and muscle relaxant weaning

After admission, HFNC with 45 liters flow rate and 75% FiO_2_ was applied, and the patient got respiratory stabilized. In the evening, due to deterioration and high oxygen requirements, the patient had to be intubated. The obstetric team regularly followed up with the patient. Clinically, the patient showed a full picture of severe ARDS. COVID-19 pneumonia has been treated as per local protocol (Table 4). The patient required simultaneously up to four analgesic and sedative agents at high doses (Figure 2) plus NMBA cisatracurium up to 3 mcg/kg/min to maintain oxygenation and ventilation. This challenged the weaning from sedation and ventilation accordingly. Taking into account the experience with the first case, a multidisciplinary decision for bedside tracheostomy has been made. On day 8 of ICU admission, when tracheostomy was applied, she required continuous infusions of fentanyl 5 mcg/kg/hour, propofol 45 mcg/kg/min, and midazolam two-point 5 mcg/kg/min to maintain Richmond Agitation Sedation Scale (RASS) of minus two. Ventilator settings early on the day of tracheostomy were APV-CMV, RR 25/min, tidal volume 360 mL, PEEP 12 cmH_2_O, FiO_2_ 45%. The bedside percutaneous dilatative tracheostomy under bronchoscopy guidance was performed without any complications, and tracheostomy tube size eight was inserted. COVID-19 PCR status two days after the procedure was negative. On the second day after the tracheostomy, the patient developed paralytic ileus with severe abdominal pain, treated conservatively. The weaning from analgesia and sedation took about four days because of the ileus (Figure 2). Subsequent weaning from the ventilator went smoothly as well, and the patient was decannulated eight days after tracheostomy. On the following day, she was transferred to the ward after 17 days of ICU stay and discharged home after two more days, with in total of 20 days of hospital stay. Follow-up by primary care physician and obstetrician in the next two months did not show any serious concerns. Patient delivered per emergency CS in week 38 + one without any complications. The baby has been discharged home with the mother in good condition.

**Table 4:** COVID-19 pneumonia treatment

Drug	Dose	Duration
Piperacillin/Tazobactam	4,5 g, q8hr	7 days
Enoxaparine	40 mg, twice daily	10 days
Dexamethason	8 mg, daily	5 days
Mehtylprednisolon	40mg, q8hr	3 days
Remdesivir	1^st^ day 200 mg, then 100 mg, daily	4 days
Tocilizumab	400 mg, once	on two occasions
Meropenem	1 g, q8hr	5 days

## Discussion

Sedation is paramount in all patients who require MV. Adequately managed sedation prevents patient-ventilator dyssynchrony, accidental extubation, and circuit disconnections. In addition, it allows to facilitate prone ventilation and considers paralysis in those who develop refractory hypoxia [18].

The physiological and pharmacokinetic changes during pregnancy, which include an increase in blood volume, cardiac output, and total body water, may explain the requirement for high doses of sedatives and analgesics [19,20]. These factors may lead to an increase in the volume of distribution (Vd). Additionally, glomerular filtration rate and total drug clearance (Cl) are increased in pregnancy. Both increases in Vd and Cl lead to a decrease in serum drug concentration [20,21].

In this paper, we report the clinical outcomes of two pregnant patients with severe COVID-19 pneumonia after tracheostomy. Our observation was a significant reduction in sedation and pain medication after tracheostomy. We were able to wean off the sedation and analgesia completely within 48 hours of tracheostomy in the first case and within 72 hours in the second case. Both our patients required high doses of analgesics, sedatives, and an NMBA. Drug safety, possible side effects, and unpredictive maximum dose due to physiology and pharmacokinetic changes during the pregnancy have contributed to our decision for the early tracheostomy in the second case in particular.

It has been reported that critically ill COVID-19 patients tend to require higher doses of sedation and analgesia [18]. This exposes them to serious side effects, especially in pregnant patients, such as hypotension and bradycardia, which may further affect placental perfusion, prolonged duration of MV, affect neurological assessment, cause paralytic ileus, delirium, nausea, and vomiting [22]. Furthermore, prolonged periods of sedation may lead to drug accumulation, tolerance, and tachyphylaxis.

In a prospective cohort study of 164 patients, tracheostomy was associated with significantly higher 30-day survival among COVID-19 pneumonia patients requiring MV [23]. Moreover, early tracheostomy (within 14 days from intubation) was associated with a decrease in both ventilator days and ICU length of stay, although no statistically significant difference in decannulation time was observed between early and late tracheostomy (13 versus 15 days, respectively). In this study, sedative agents were weaned off within 48 hours in 76% of the tracheostomized patients.

Carmichael et al. were able to liberate 81% of tracheostomized COVID-19 pneumonia patients from the ventilator an average of nine days post-tracheostomy. Additionally, sedation and pain medication requirements decreased significantly within one week after the procedure [16]. Although this study observed a significant decrease in sedation requirement and ventilator days, the authors did not compare these findings with non-tracheotomized patients. They did not mention if pregnant patients were included in this study.

During the COVID-19 surges, when most ICUs were fully occupied, reducing the patient's stay in the ICU is considered one of the most valuable outcomes to empty this bed for new admissions. Any intervention that contributes to this objective should be considered. In a recently published meta-analysis of 14 non-randomized studies, early tracheostomy was associated with a decrease in mortality risk and ICU stays [24]. These findings should be interpreted with caution, due to the high heterogeneity between the included studies (I^2^ = 87% and 70% in mortality, and ICU stay, respectively).

## Conclusions

Pregnant patients with severe COVID-19 pneumonia seem to require high doses of analgesics, sedatives, and NMBAs. A prolonged period of sedation and ventilation, and possible drug side effects, are serious concerns that should be taken into account. Early tracheostomy can be considered a strategy to facilitate weaning from MV in intubated pregnant and early postpartum COVID-19 patients requiring high doses of sedation and analgesia. However, prospective studies are required to confirm these findings.
